# Noninvasive Ventilation in Treatment of Respiratory Failure-Related COVID-19 Infection: Review of the Literature

**DOI:** 10.1155/2022/9914081

**Published:** 2022-08-31

**Authors:** Bushra Mina, Alexander Newton, Vijay Hadda

**Affiliations:** ^1^Division, Pulmonary Critical Care Medicine, Lenox Hill Hospital, New York, NY, USA; ^2^Department of Internal Medicine, Lenox Hill Hospital, New York, NY, USA; ^3^Department of Pulmonary, Critical Care & Sleep Medicine, All India Institute of Medical Sciences, New Delhi, India

## Abstract

The recently diagnosed coronavirus disease 2019 (COVID-19), caused by severe acute respiratory syndrome coronavirus 2 (SARS-CoV-2), in December 2019 commonly affects the respiratory system. The incidence of acute hypoxic respiratory failure varied among epidemiological studies with high percentage of patients requiring mechanical ventilation with a high mortality. Noninvasive ventilation is an alternative tool for ventilatory support instead of invasive mechanical ventilation, especially with scarce resources and intensive care beds. Initially, there were concerns by the national societies regarding utilization of noninvasive ventilation in acute respiratory failure. Recent publications reflect the gained experience with the safe utilization of noninvasive mechanical ventilation. Noninvasive ventilation has beneficiary role in treatment of acute hypoxic respiratory failure with proper indications, setting, monitoring, and timely escalation of therapy. Patients should be monitored frequently for signs of improvement or deterioration in the clinical status. Awareness of indications, contraindications, and parameters reflecting either success or failure of noninvasive ventilation in the management of acute respiratory failure secondary to COVID-19 is essential for improvement of outcomes.

## 1. Introduction

The recently diagnosed coronavirus disease 2019 (COVID-19), caused by severe acute respiratory syndrome coronavirus 2 (SARS-CoV-2), was initially recognized in Wuhan City, Hubei Province, China, in December 2019. The WHO declared COVID-19 as a global health emergency on January 31, 2020, and subsequently, a pandemic on March 11, 2020. Globally, the disease has been reported in more than 210 countries with 512,225,941 confirmed cases and 6,230,957 mortalities till April 28, 2022. Since the first wave of COVID-19, many countries have already seen the third wave of the spread of this virus (e.g., India, Germany, USA, and others), and a few countries (e.g., India, South Africa, and Zimbabwe) are now witnessing the fourth and higher waves of the COVID-19 pandemic. Different variants of the SARS-CoV-2 virus have been identified since the initial pandemic. The variants are different in rate of infectibility and fatality. The Omicron variant is more infectious than the Delta variant but less fatal, and consequently, less hospitalization.

Disease manifestation varies from mild flu like symptoms to severe respiratory failure with multiple organ involvement. The risk of death among individuals infected with COVID-19 was found to be in the range of 0.3% to 0.6%, which is comparable to that of a previous Asian influenza pandemic (1957 to 1958) [1]. It is now well recognized that the severe pulmonary involvement manifested as acute respiratory failure and adult respiratory distress syndrome is strongly associated with worse outcomes.

### 1.1. Pulmonary Manifestations of COVID-19

The respiratory system is the most common organ affected by the COVID-19. The common symptoms of the COVID-19 include fever (82–91%), cough (57–72%), dyspnea (21–45%), and sputum production (26–28%) which are usually mild [2, 3]. In severe cases, the clinical course can progress to pneumonia with hypoxic respiratory failure and acute respiratory distress syndrome (ARDS).

There are studies which have described the patterns of lung involvement on computer tomography (CT) scan of the thorax by COVID-19. In fact, CT findings can be diagnostic of COVID-19 among patients with RT-PCR-negative patients. The common radiological abnormalities on CT scans included ground glass opacity (14–98%), consolidation (2–64%), consolidation plus GGO (19–59%), interlobular septal thickening (1–75%), reticular pattern (1–22%), crazy paving (5–36%), air bronchogram (21–80%), and bronchial wall thickening (11–23%) [4].

### 1.2. Incidence of Acute Respiratory Failure (ARF)

Incidence of acute respiratory failure and need for mechanical ventilation varied among published studies due to different timings in the pandemic, among countries, age groups, resources, and variation in severity of disease at time of presentation [5]. Acute hypoxic respiratory failure was defined as (1) respiratory rate of 30 breaths per minute or greater; (2) oxygen saturation of 93% or less in a resting state; (3) arterial oxygen tension (PaO_2_)/inspiratory oxygen fraction (FiO_2_) of 300 mm·Hg or less (1 mm·Hg = 0.133 kPa); or (4) need for mechanical ventilation [6]. A study from China reported that 5.0% of patients required admission in the ICU and 2.3% underwent invasive mechanical ventilation [2]. Another epidemiological study reported 25% of patients with severe or critical disease required mechanical ventilation [7]. In USA, 2.3% of hospitalized patients were admitted to ICU [8].

ARDS, the most severe for hypoxemia, is defined according to Berlin definition into mild, moderate, and severe, depending on the degree of PaO_2_/FiO_2_ ratio [9]. The frequency of ARDS in COVID-19 varied between studies. A retrospective analysis reported incidence of ARDS of 74.1%, whereas Lai and colleagues identified that among hospitalized patients, about 20% developed ARDS and >25% of patients required ICU admission [10, 11]. A meta-analysis of observational studies and case reports has reported an incidence of ARDS as high as 32.8% of patients during their hospital admission [12]. Another study by Tzotzos et al. and colleagues reported the weighted averages for the incidence of ARDS among published studies. They reported that among hospitalized patients, approximately 33% developed ARDS, 26% required ICU admission, 16% received invasive mechanical ventilation, and 16% died. They also reported that two-third (63%) of patients who required ICU admission received mechanical ventilation; the indication of mechanical ventilation was ARDS in 75% of patients. COVID-19-associated ARDS mortality rate was 40% and 59% among who received invasive mechanical ventilation (IMV) [13].

### 1.3. Pathophysiology of COVID-19 ARDS

Marini described two distinct subsets of ARDS in COVID-19 patients. “Type L” early in the process of the disease is related to interstitial rather than alveolar edema with relatively good compliance despite poor oxygenation, low elastance, lower lung weight, and low response to PEEP. That stage can progress to stage “type H” (similar to typical ARDS) with poor compliance, higher elastance, higher lung volume, and low response to PEEP. “Type L” ARDS stabilizes easily with just increasing the FiO_2_ and may benefit from high flow nasal cannula or noninvasive ventilation (NIV) depending on the respiratory drive. Targeting a lower PEEP (8–10 cm H_2_O) is recommended for “type L” ARDS to avoid ventilator-induced lung injury (VILI) and avoiding patient self-induced ventilator lung-induced injury (P-SILI). Larger tidal volume can be applied for “type L” (7–8 ml/kg ideal body weight). Lung protective ventilation protocol should be applied to “type H” ARDS with low tidal volume (6 ml/kg ideal body weight) and higher PEEP (<15 cm H_2_O) [14].

Invasive mechanical ventilation (IMV) is essential therapeutic modality in the management of acute respiratory failure but is associated with potentially preventable complications such as atelectrauma, barotrauma, volutrauma, biotrauma, and infection [15, 16]. Use of NIV may reduce many of these complications without adversely affecting the outcomes. Currently, NIV has been recommended for the treatment of ARF due to acute exacerbation COPD, acute cardiogenic pulmonary edema, in immunocompromised patients, and de novo ARF [17]. De novo respiratory failure is defined as respiratory failure occurring without prior chronic respiratory disease with significant hypoxemia (PaO_2_/FiO_2_ ≤ 200), tachypnea (respiratory rate >30–35 breaths/min), and a non-COPD diagnosis (e.g., pneumonia and/or ARDS). NIV can decrease mortality (RR 0.83, 95% CI 0.65–1.05) and the need for intubation (*RR* 0.75, 95% CI 0.63–0.89). Patients should be carefully selected, closely monitored in the ICU, and reassessed early after starting NIV for evidence of worsening respiratory failure and escalation to invasive mechanical ventilation [17].

ARF secondary to COVID-19 remains a serious cause of morbidity and mortality as we are experiencing the fourth wave of COVID-19 with mutated variant with a different infectivity. Despite significant increase in our understanding of the pathophysiology of ARDS in COVID-19, the best pharmacological and nonpharmacological therapeutic modalities for this disease are yet not known. However, the management of hypoxemia is pivotal for good outcome. As critical care resources remain scarce, NIV remains a tool that can be utilized in the treatment of ARF in COVID-19. Recent publication addressed previous concerns in regard to utilization of NIV in treatment of ARF related to COVID-19[18–23]. However, there are still many unanswered questions. The objective of the article is to review the current literature and explore the effectiveness and safety of NIV in treatment of COVID-19-related acute hypoxic respiratory failure.

## 2. Methodology

The literature review was focused on the topics of NIV and treatment of ARF in COVID-19 with critical analysis of the data for exploring the strengths and weaknesses. We complete an extensive literature search to ensure a comprehensive review of existing studies on the topic of our document. We identified new and additional research by performing targeted keyword searches (NIV, acute respiratory failure, COVID-19, and pulmonary complications of COVID-19) through PubMed and Google Scholar. We identified and selected a total of 68 peer-reviewed, scholarly sources, in particular the research topics, as our guide. For the literature review, we ensured discussion of all literature is presented in past tense.

## 3. Results

### 3.1. Application of NIV

Initially, the indications and contraindications of NIV in COVID-19 were extrapolated from the general recommendation of NIV in the management of ARF and published literature from previous pandemic (such as H1N1) [18]. The recent literature provides data related to the application of NIV which is COVID-19-related ARF [24–28]. Recommended criteria for application of NIV in selected patients are shown in Table 1.

NIV is contraindicated under certain situations in COVID-19 patients as listed in Table 2.

### 3.2. Modalities, Interfaces, and Settings of NIV

Noninvasive ventilation can be in the form of bilevel NIV or CPAP primarily with escalation to NIV. Among interfaces, full-face mask or helmet (preferable) is recommended but not nasal masks. Monitor for full fit of the full-face mask and any evidence of air leak. Recommended settings for NIV are given as follows:

- CPAP for hypoxemic respiratory failure, commencing at 10 cm H_2_O pressure and an FiO_2_ of 0.6, with potential to increase to 12–15 cm H_2_O and FiO_2_ 1.0
- BiPAP may be used for hypercapnic acute on chronic respiratory failure
- Recommended high peep (8–12 cm H_2_O) and low-pressure support in order to obtain tidal volume <9 ml/kg ideal body weight
- Titrate FiO_2_ to achieve target SpO_2_ 94–96% or 88–92% for patients with acute on chronic respiratory failure

### 3.3. Monitoring Response to NIV

Response to NIV should be monitored every 1–2 hours for either improvement or deterioration in the respiratory and clinical status. It is prudent to identify patients for potential failure of NIV and escalation to mechanical ventilation, without delay in endotracheal intubation. Also, the patient should be monitored for possible mask intolerance and mask malposition, with possible air leak with limitation of PEEP and decruitment leading to deterioration is gas exchange and increase in work of breathing [17, 19–23, 29–33].

The following parameters should be monitored as a standard practice:

- Oxygen saturation or arterial blood gas analysis
- Tidal volume
- Respiratory rate
- Accessory respiratory muscles
- Hemodynamics (blood pressure, heart rate, arrhythmias)
- Mental status
- Gastric distension and aspiration risk
- Organ failure
- Noncompliance

### 3.4. Indications of NIV Failure

The incidence of NIV failure in moderate and severe ARDS is reported in >50% of cases, with almost 50% mortality rates [31]. Indicators of NIV failure include deterioration of clinical and respiratory status, worsening of oxygenation with increase in respiratory effort, within 1–2 hour of initiation of NIV (Table 3) [29, 34–37]. ROX index is a useful tool to guide physicians in treating patients with moderate acute respiratory failure especially in a non-ICU setting. A ROX value <5.99 was associated with an increased risk of failure (*p*=0008 log-rank test) [38].

Factors that are associated with increased mortality with NIV are moderate and severe ARDS, simplified acute physiology score [SAPS] >37, degree of hypoxemia with PaO_2_/FiO_2_ ratio <150 mm·Hg, high tidal volumes (>9.2 or 9.5 mL/kg), presence of bilateral pneumonia, and progressive worsening of the chest CT scan [10, 29, 34–40].

High tidal volumes (>9.2 or 9.5 mL/kg) under NIV are associated with increased mortality related to high spontaneous respiratory drive, with high volume resulting in transpulmonary pressure variation which can lead to volutrauma and patient self-induced lung injury (P-SILI) [34–37, 41, 42].

### 3.5. Effectiveness of NIV

Faranone et al. assessed the effectiveness and safety of NIV in treatment of acute hypoxemic respiratory failure (AHRF) among 50 patients with COVID-19. Authors reported a success rate of 64% among who received NIV without limitation. Successful weaning from NIV was predicted by use of corticosteroids (OR 15.4, CI 1.79–132.57; *p*=0.013) and the increase in the PaO_2_/FiO_2_ ratio measured 24–48 h after NIV initiation (OR 1.02, CI 1–1.03; *p*=0.015), while it was inversely correlated with the presence of a DNI order (OR 0.03, CI 0.001–0.57; *p*=0.020) [43]. Menzella et al. evaluated outcomes of 79 patients who required NIV for AHRF secondary to COVID-19 infection. NIV was successful in 48.1%, and 25.3% required invasive mechanical ventilation after a trial of NIV of whom 57% were discharged alive. The authors concluded that NIV can be applied safely, and invasive mechanical ventilation can be avoided in 50% of cases [44]. In another study, authors reported that heart rate, acidosis (assessed by pH), consciousness (assessed by GCS), oxygenation, and respiratory rate (HACOR) at 1 hr were independent risk factors for NIV failure. The HACOR ranged from 1 to 25, and each point increase in score was associated with odds ratio (OR) of NIV failure 1.73 (95% CI 1.58–1.95) [39].

NIV (CPAP) has been compared with high flow oxygen by nasal cannula (HFNC) and conventional oxygen for management of AHRF due to COVID-19 [45]. The study reported a significant reduction in the need for mechanical ventilation among patients managed with CPAP. Escalation to invasive mechanical ventilation was significantly lower with CPAP (36.3%) vs. conventional oxygen therapy (44.4%) (absolute difference, −8% [95% CI, −15% to −1%]; *p*=0.03). ICU admission was less in the CPAP group compared with the conventional oxygen therapy group (55.4% vs. 62.9%, respectively: absolute difference, −7% [95% CI, −15% to −3%]). CPAP, compared to conventional oxygen therapy, was associated with more frequent adverse events in 34.2% vs. 13.9%, respectively [45].

Helmet noninvasive respiratory support has been suggested as alternative to avoid droplet dispersion and healthcare worker contamination. The benefit of CPAP application by means of helmet can improve patient comfort level and increase tolerability. The CO_2_ rebreathing is of concern and depends on two factors: the fresh gas passing through the helmet and the amount of CO_2_ produced by the patient. The recommendation is to initiate CPAP at 5 cm H_2_O and to titrate according to blood gas analysis and respiratory mechanics. PEEP should not exceed 12–13 cm H_2_O in order to avoid VAE and effect on hemodynamic due to increase in intrathoracic pressure. Weaning from the helmet should be initiated by incremental decrease in PEEP while maintaining PO_2_/FiO_2_ ratio with FiO_2_ not higher than 50%. A proposed algorithm for the management of helmet CPAP in ARF was recently published [26].

A recent review on mortality and clinical outcomes of patient with COVID-19 pneumonia treated with NIV concluded that CPAP and NIV appeared equally and frequently applied in patients with COVID-19 pneumonia but associated with higher mortality. Utilization rate of CPAP and NIV was 48.4% and 46%, respectively. Noninvasive respiratory support was unsuccessful in 47.7%, of which 26.5% were intubated with 40.9% mortality. NIV was associated with a higher in-hospital mortality compared to CPAP (35.1% vs. 22.2%). The indications for endotracheal intubation and invasive mechanical ventilation were decreased level of consciousness, exhaustion, refractory hypoxemia, sepsis, and hemodynamic instability [46].

Factors that are associated with increased mortality with NIV are moderate and severe ARDS, simplified acute physiology score [SAPS] >37, degree of hypoxemia with PaO_2_/FiO_2_ ratio <150 mm·Hg, high tidal volumes (>9.2 or 9.5 mL/kg), presence of bilateral pneumonia, and progressive worsening of the chest CT scan [10, 29, 34–37, 41, 42]. Reported total mortality rate ranged between 24.6% and 25.3% [45, 47]. Chacko et al. reported overall mortality in patients who required NIV was 30.1%. On adjusted analysis, mortality was associated with older age (OR, 1.08; 95% CI, 1.04 to 1.12), severe ARDS (OR, 4.04; 95% CI, 1.08 to 15.1), and higher peak D-dimer level (OR, 2.75; 95% CI, 1.19 to 6.37), requirement for intubation (OR, 9.36; 95% CI, 3.38 to 25.94), and need for inotropes and/or dialysis (OR, 9.19; 95% CI, 2.83 to 29.9) (3.1%) [48].

### 3.6. Alternatives to NIV

HFNC can be utilized safely in acute hypoxic respiratory failure associated with severe COVID-19 pneumonia. A prospective study from two tertiary care hospitals evaluated the incidence of successful weaning from HFNC as a primary outcome; in addition, study reported the incidence of failure and need for escalation and endotracheal intubation, and overall mortality. Study showed that HFNC was successful in 47% and 93% of patients who were discharged home. Predictors of success of HFNC, at the time of application, were higher oxygen saturation, lower respiratory rate, lower oxygen requirement within 6 hours of HFNC, higher ROX-6 and mROX-6 score, and no steroid usage. The authors concluded that HFC was feasible in the treatment of AHRF associated with severe COVID-19 pneumonia, but mortality was high in patients who failed HFNC trial [49].

HFNC can improve dyspnea scores in patients with AHRF and be applied in non-ICU areas [50–52]. In a prospective randomized trial, HFNC was compared to conventional oxygen therapy (COT) in the treatment of hypoxic respiratory failure. HFNC significantly improved dyspnea (2.0 ± 1.8 vs. 3.8 ± 2.3, *p*=0.01) compared with COT. The HFNC decreased the respiratory frequency within 5 minutes of its application. Roca et al. reported improvement in dyspnea (*p*=0.001) and overall comfort (*p* < 0.001) with HFNC compared to conventional face mask 50 [51]. HFNC can be utilized during breaks from NIV with significantly lower dyspnea scores compared to standard oxygen therapy [53]. HFNC may be an alternative method for palliative patients with hypoxic respiratory failure and do-not-intubate status in improving dyspnea within the first hour of treatment [54].

### 3.7. Healthcare Worker Risks and Environmental Protections

Safety of the delivery of the ventilatory support is one major concern for healthcare worker regarding bio-aerosolization and nosocomial SARS-CoV-2 transmission. Menzella and Avdeev et al. reported low risk of healthcare worker contracting COVID-19 infection (1.6%) [44, 55].

Invasive ventilation and helmet ventilation with a PEEP valve were found to be associated with the lowest bacteriophage concentrations in the air, and HFNO and nasal prongs were associated with the highest concentrations in the environment [56]. In another study performed on healthy subjects, neither humidified HFNC nor NIV increased aerosol generation from the respiratory tract measured in a negative pressure room. Aerosol generation is influenced more by breathing pattern and coughing [57]. Personal protective equipment and environmental control (negative pressure rooms) should be the initial concern and consideration when managing patients with COVID-19. There should be emphasis on adherence with infection control protocols among healthcare workers to decrease the incidence of infection [47].

### 3.8. Application of NIV outside of ICU Settings

There was an increase in the utilization of NIV outside the ICU area due to lack of ICU resources and bed availability. Nava reported feasibility of out-of-ICU noninvasive respiratory support in the treatment of patients with COVID-19 pneumonia and favorable outcomes. Majority of patients were treated with CPAP. The 30-day unadjusted mortality was 30% for both CPAP and NIV. Incidence of endotracheal intubation for CPAP and NIV was 25% and 28%, respectively. Mortality was related to age and comorbidities but not to noninvasive respiratory support after adjustment for cofounders. There was 11.4% incidence of healthcare workers tested positive for infection [58].

In a prospective single-day observational study from 31 hospitals in Lombardy, Italy, 10% of patients received NIV outside the ICU, and 68% were treated with CPAP delivered by helmet. Failure rate was 37.6%; on the contrary, 62.4% patients were discharged alive without need for intubation. In-hospital mortality was 25% [59].

NIV can be applied on regular wards as a viable ceiling treatment option in patients with underlying severe comorbidities, such as CAD and hematological diseases, with acute hypoxic respiratory failure secondary to severe COVID-19 pneumonia. Reported survival rate was 29%. Worsening hemodynamic and vital signs within 48 hours of initiating NIV were poor indicators [60].

## 4. Discussion

Around 5% of COVID-19 patients develop a critical form of the disease with AHRF necessitating ICU admission, and delaying intubation may prove fatal among these patients. Adding to the controversy, early intubation, and mechanical ventilation, within 2 days of ICU admission, for patients with COVID-19 with AHRF was associated with increased 60-day mortality as compared to initial use of noninvasive oxygen support [42.7% versus 21.9% (*p* < 0.01)]. In addition, delayed intubation group (intubation after the first 2 days after ICU admission) had a similar outcome to those in the early IMV group, with a 60-day mortality of 42.2% and 42.7%, respectively. Patients without any IMV intervention had the highest survival rate with 60-day mortality of 10.8% [61].

Data for the Lombardy region in Italy support the above data. Patients who subsequently intubated after a trial of unsuccessful NIV had a significantly lower chance of survival compared with the patients who continued NIV and did not require IMV (HR, 1.69; 95% CI, 1.43–1.98; *p* < 0.001). The mortality of the patients who undergone subsequent intubation was similar to the group of patients who were treated with IMV at the time on admission to ICU (HR for IMV vs. NIV failure, 1.20; 95% CI, 0.95–1.53; *p*=0.12). The mortality rate and mortality rate per 1000 patient-days were lower in the NIV group compared to IMV group [62].

Data from the interim analysis of the international, multicenter HOPE COVID-19 registry concluded that NIV can be a feasible alternative to IMV especially when ICU resources are limited. NIV was indicated in 20% of their study population. NIV was successful in 50% of cases. In-hospital death was 37.7%, while 15.9% of patients needed invasive ventilation and were associated with high rate of in-hospital death. Those requiring invasive ventilation had the lowest survival rate (41.9%). Both NIV and IMV groups were associated with an increased risk of mortality (HR 1.26, 95% CI 1.04 to 1.53 and HR 1.91, 95% CI 1.45 to 2.53, respectively). The population treated with IMV at any point had increased mortality risk compared to those who only received NIV (HR 1.52, 95% CI 1.11 to 2.06, *p*=0.008). 37.7%) [63].

Recommendations and consensus statements by medical societies (NIH, Australian and New Zealand Intensive Care Society, WHO, Surviving Sepsis Guidelines SCCM, and Austrian Society of Pneumatology) [20–23, 31] regarding NIV in management of ARF have been published based on published studies with variability of level of evidence, absence of randomization, and a different methodology. Most observational studies suggested that NIV can be utilized with caution in selected patients with ARF, in particular mild ARDS, and to be treated under close observation and readiness to escalate to IMV. NIV could be utilized to avoid intubation or re-intubation postextubation. Ideally, NIV should be applied in negative pressure rooms with minimum six air exchanges per hour or 12 as recommended by WHO, or in a single occupancy neutral pressure room (if negative pressure room is unavailable) with proper adherence to wearing personal protective equipment (PPE) [18–23, 29–33].

Different medical societies were cautious in their recommendations for high flow nasal cannula (HFNC) and noninvasive mechanical ventilation (NIV) in the management of acute respiratory failure related to COVID-19, especially, in the absence of indication for endotracheal intubation and mechanical ventilation. The concern is delaying endotracheal intubation and increasing mortality. Data from non-COVID-19 trials showed reduction in the requirement of invasive mechanical ventilation when HFNC or NIV, compared to conventional oxygen therapy, was utilized with decrease in the endotracheal intubation rates and escalation of respiratory support [64, 65]. NIH recommended HFNC oxygen over NIV; NIV is recommended only in controlled setting when HFNC is unavailable. Panel also recommended the close monitoring of patients for worsening of the respiratory failure and avoiding delay intubation [66].

Several factors contribute to failure of NIV such as type of interface, ventilatory modality (i.e., continuous positive airway pressure (CPAP) vs. bilevel), and lower or higher positive pressures, and pathophysiological characteristics of COVID-19-related interstitial pneumonia and ARDS [14, 67].

NIV is an option for respiratory management of COVID-19-related acute hypoxic respiratory failure. Proper selection of patients, application of proper setting with fitting of face mask or helmet in proper setting, close monitoring for elements of worsening of respiratory status, and readiness for escalation of care are essential in the management of those patients.

## 5. Conclusion

NIV is feasible in the treatment of AHRF secondary to COVID-19 infection both in the ICU and out-of-ICU setting. NIV is expected to improve oxygenation and decrease the work of breathing. It can reduce the need for mechanical ventilation and complications associated with it. Helmet noninvasive respiratory support is as alternative to oronasal/full-face mask during NIV. Close monitoring and early identification of NIV failure are key to avoid delayed intubation-associated mortality. Well-designed studies are needed to find the best protocol, including initial settings and weaning, and interface to be used. Also, further studies are required to define the exact role of NIV, especially when it is compared with HFNC.

## Figures and Tables

**Table 1:** Criteria for application of NIV in selected patients.

(1) Clinical criteria:
(i) Moderate to severe dyspnea with signs of respiratory effort and use of accessory muscles or paradoxical abdominal movement (increase work of breathing) or staccato speech.
(ii) Tachypnea over 30 bpm.
(iii) No multi-organ failure (APACHE<20)
(iv) Known patient history of OSA, COPD, congestive heart failure, or cardiogenic pulmonary edema and neuromuscular disorders with acute or exacerbated hypercapnic respiratory failure.
(v) Availability of an expert team and continuous monitoring.
(vi) Early intubation (within the hour) if there is no improvement.
(vii) Patients with do-not-intubate status.
(viii) Postextubation phase of ARDS.

*(2) Blood gas criteria*:
(i) Need for FiO_2_ greater than 0.4 to achieve an SpO_2_ of at least 92%, or SpO_2_ <94%, *RR* > 20 with poor response to oxygen 10–15 l/min.
(ii) Acute hypercapnic respiratory failure (pH < 7.35 with PaCO_2_ > 45 mm·hg).
(iii) PaO_2_/FiO_2_ > 150 but <300, or SpO_2_ < 90–94% on non-rebreather.

**Table 2:** Contraindications for NIV in COVID patients.

Indication for invasive mechanical ventilation
Limited personnel experience with HFNC/NIV
Lack of capability of monitoring
Lack of infectious control and control of aerosolized transmission
Hemodynamic instability and cardiac arrhythmias
Multiple organ failure
Abnormal mental status or encephalopathy
Over-ventilation and “patient-induced lung injury” (PILI)
Cardiopulmonary arrest
Uncooperative patients
Inability to protect airways
Anatomical and/or subjective difficulties gaining access to the airway
Gastrointestinal bleeding, ileus, or risk for aspiration
Severe hypoxemia or acidosis (pH < 7.1)
Excessive secretions
Recent upper airway or upper gastrointestinal surgery
Severe hypoxemia on admission defined as PaO_2_/FiO_2_ < 150
Pneumothorax, pleural effusion, or pulmonary embolism
Recent facial trauma or facial surgery
SOFA score >5 is predictive of NIV failure
CXR/CT showing evidence of bilateral, multi-lobar involvement

**Table 3:** Indicators of NIV failure.

Simplified acute physiology score [SAPS] >37
High APACHE score
PaO_2_/FiO_2_ ratio <150 mm·Hg
High tidal volumes (>9.2 or 9.5 mL/kg)
Respiratory rate >30/min
HACOR score >5 [34]
Acute respiratory acidosis with rise in PaCO_2_
ROX index <3 at 2 hours

## Data Availability

The review article data were obtained from PubMed.
